# Heat-Killed *Lactobacillus brevis* Enhances Phagocytic Activity and Generates Immune-Stimulatory Effects through Activating the TAK1 Pathway

**DOI:** 10.4014/jmb.2002.02004

**Published:** 2020-07-19

**Authors:** Minju Jeong†, Jae Hwan Kim†, Ji Su Lee, Shin Dal Kang, Sangmin Shim, Moon Young Jung, Hee Yang, Sanguine Byun, Ki Won Lee

**Affiliations:** 1 Department of Agricultural Biotechnology, Seoul National University, Seoul 08826, Republic of Korea; 2 Division of Bioengineering, Incheon National University, Incheon 22012, Republic of Korea; 3 Research Institute of Food and Biotechnology, SPC Group, Seoul 151742, Republic of Korea; 4 Advanced Institutes of Convergence Technology, Seoul National University, Suwon 16229, Republic of Korea; 5 Department of Biotechnology, Yonsei University, Seoul 03722, Republic of Korea; 6 Center for Food and Bioconvergence, Seoul National University, Seoul 08826, Republic of Korea

**Keywords:** *Lactobacillus brevis*, probiotics, immune stimulation, TAK1, phagocytosis

## Abstract

There is an increasing interest in using inactivated probiotics to modulate the host immune system and protect against pathogens. As the immunomodulatory function of heat-killed *Lactobacillus brevis* KCTC 12777BP (LBB) and its mechanism is unclear, we investigated the effect of LBB on immune response based on the hypothesis that LBB might exert stimulatory effects on immunity. In the current study, we demonstrate that administration of LBB can exert immune-stimulatory effects and promote clearance of foreign matters through enhancing phagocytosis. Treatment with LBB induced the production of TNF-α, IL-6, and nitric oxide in macrophages. Importantly, LBB directly increased the phagocytic activity of macrophages against bacterial particles. LBB was able to promote the production of TNF-α in bone marrow-derived macrophages and splenocytes and also increase the proliferation rate of splenocytes, suggesting that the immune-stimulating activity of LBB can be observed in primary immune cells. Investigation into the molecular mechanism responsible revealed that LBB upregulates TAK1 activity and its downstream ERK, p38, and JNK signaling pathways. To further confirm the immunomodulatory capability of LBB in vivo, we orally administered LBB to mice and assessed the effect on primary splenocytes. Splenocytes isolated from LBB-treated mice exhibited higher TNF-α expression and proliferative capacity. These results show that heat-killed *L. brevis*, a wildly consumed probiotic, may provide protection against pathogens through enhancing host immunity.

## Introduction

Recently, a wide range of bioactivities have been attributed to the consumption of probiotics including protection against pathogens [1], maintenance of the intestinal barrier [2] and immunomodulatory effects [3]. Probiotics are now widely used in food manufacturing industries and as dietary supplements for health benefits [4]. According to the United Nations Food and Agriculture Organization (FAO) and the World Health Organization (WHO), probiotics are defined as “live microorganisms which when administered in adequate amounts confer a health benefit on the host.” [5] However, some studies have revealed that inactivated probiotics may carry fewer risks than live probiotics. For example, it has been reported that the infant intestinal microbiome could be adversely affected by the intake of probiotics, which could cause allergic responses in early life [6]. Furthermore, studies have demonstrated that some bifidobacteria and lactobacilli carry antibiotic resistance bringing a risk of transfer to pathogenic strains [7, 8]. Probiotics are typically manufactured to have less antibiotics resistance for safety reasons [9]. Such antibiotic resistance within probiotics can be removed by heat treatment [10]. Other treatments, such as ultraviolet light and sonication, have been also applied to probiotics for inactivation [11].

Heat-killed probiotic cultures have been reported to modulate immune responses. For instance, heat-killed *Lactobacillus* strains increase mouse splenocyte proliferation and stimulate Th1 immune responses [12]. Several strains of heat-killed *Lactobacillus* have been shown to enhance tumor necrosis factor-α (TNF-α) production in RAW264.7 macrophage cells and mouse splenocytes via upregulation of c-Jun N-terminal kinase (JNK) [13]. Heat-killed *Lactobacillus plantarum* has been reported to stimulate phagocytic activity and elicit immunomodulatory effects through upregulating Signal Transducer and Transcription (STAT) and Mitogen Activated Protein Kinase (MAPKL) pathways [14]. In a virus infection model, administration of heat-killed *Lactobacillus casei* led to higher survival rates and lower weight loss compared to non-treated groups via the downregulation of lung viral loads and inflammatory cytokines production [15].

Macrophages play an essential role in innate immunity by providing protection against invading pathogens [16]. Phagocytic activity by macrophages is an important component of the innate immune system. Phagocytosis regulates tissue homeostasis and immunity [17] by clearing pathogens and removing dead cells [16, 18]. TNF-α and interleukin-6 (IL-6) participate in controlling the host defense system and are secreted by activated macrophages. Activated macrophages can also contribute to the regulation of tissue homeostasis through the secretion of enzymes, proteinases and nitrogen/reactive oxygen species [16, 18, 19]. Mitogen-activated protein kinase kinase kinase 7 (TAK1), a member of the MAP3K family [20], is a essential kinase in the activation of macrophages [21]. Also, the MAPK pathway, which includes JNK, extracellular signal–regulated kinases-1/2 (ERK-1/2) and p38, is involved in macrophages activiation [22].

*L. brevis* can be found in various types of foods and plants, and also in the human microbiome [23-25]. Previous studies have investigated the bioacitive properties of heat-killed *L. brevis*, which include the attenuation of alcoholic liver disease [26] and amelioratoin of atopic dermatitis [27, 28]. In addition, it has been shown that heat- killed *L. brevis* can promote skin health [29, 30] and aid in alleviating sleep disorders [31, 32]. Since LBB has been reported to display health-promoting effects in various models, we hypothesized that LBB might exert immunomodulatory effects. We sought to investigate the immune-stimulatory effects in vitro and in vivo and analyze the underlying molecular mechanisms using LBB.

## Materials and Methods

### Preparation of LBB and Heat-Killed *L. rhamnosus* GG KCTC5033 (LGG)

Heat-Killed *L. brevis* KCTC 12777BP (LBB) was isolated from sourdough fermented with a Korean traditional starter. *L. brevis* KCTC 12777BP was provided by the SPC group. To identify the species of *L. brevis* KCTC 12777BP, we conducted comparative analysis based on 16S rRNA gene sequencing (Table S1). *L. brevis* KCTC 12777BP, *L. rhamnosus* GG KCTC5033, and *L. brevis* KACC 11433 were pre-cultured in 10 ml of MRS broth supplemented with 2% maltose (mMRS) at 30°C for 24 h. Cultures of *L. brevis* KCTC 12777BP and *L. rhamnosus* GG KCTC5033 in 5 L of mMRS were prepared by repeating this cultivation process. After these incubation processes, *L. brevis* KCTC 12777BP, *L. rhamnosus* GG KCTC5033, and *L. brevis* KACC 11433 were isolated by centrifugation. Distilled water was added to isolated *L. brevis* KCTC 12777BP, *L. rhamnosus* GG KCTC5033, and *L. brevis* KACC 11433 to achieve a moisture content of 90%. Then, heat-treatment at 121°C for 15 min were performed on *L. brevis* KCTC 12777BP, *L. rhamnosus* GG KCTC5033, and *L. brevis* KACC 11433. The whole cell lysates were freeze-dried and used for experiments.

### Cell Culture

RAW264.7 murine macrophage cells were obtained from the Korean Cell Line Bank and cultured in Dulbecco’s Modified Eagle Medium (DMEM, Welgene, Korea) supplemented with 10% fetal bovine serum (Gibco, USA) and 1% penicillin and streptomycin (Corning, USA). RAW264.7 murine macrophage cells were maintained in a 5% CO_2_ humidified chamber at 37°C.

### Enzyme-Linked Immunosorbent assay (ELISA)

TNF-α and IL-6 levels in cell culture supernatant were analyzed using mouse TNF-α and IL-6 ELISA kits according to the manufacturer’s protocol (R&D Systems, USA). Briefly, 96-well microplates were coated with the capture antibody and incubated overnight at room temperature (RT). The next day, plates were blocked with 1% BSA in PBS and washed 3 times with wash buffer. 100 μl of samples or standards were added to each well and incubated for 2 h at RT. After 2 h, each well was washed 3 to 4 times with wash buffer and the corresponding detection antibody was added to the wells. After 2 h, the plates were washed 3 times and the wells were incubated with streptavidin-HRP for 20 min. After the final washing step, the substrate solution was added to each well and incubated for 20 min in the dark. HRP/substrate reaction was finished with a stop solution (2 N H_2_SO_4_), and the absorbance at 450-570 nm was measured using the Varioskan Lux Multimode microplate reader (Thermo Fisher Scientific, USA).

### Cell Viability Assay

To evaluate cell viability, RAW264.7 macrophage cells were seeded into a 96-well white luminescence plate (SPL life science Co., Republic of Korea) at a density of 4 × 10^4^ cells/well and maintained in a 5% CO_2_ humidified chamber at 37°C for 24 h. Then, the medium was changed to serum-free DMEM and incubated for another 24 h. After that, various concentrations of LBB were treated to each well. After 24 h, plates were treated by luminescence reagents for assessing cell viability according to the manufacturer’s instructions (CellTiter-Glo Luminescent Cell Viability Assay kit, Promega, USA). Plates were shaken for 1 min on an orbital shaker and incubated at RT for 10 min. Luminescence was measured using the Varioskan Lux Multimode microplate reader (Thermo Fisher Scientific).

### Phagocytosis Assay

RAW264.7 macrophage cells were uses to measure the effect of LBB on phagocytic activity. Cells were seeded into 96-well plates at a density of 4 × 10^4^ cells/well and maintained in a 5% CO_2_ humidified chamber at 37°C. After 24 h, LBB was treated to each well for 3 h, and then *E. coli* particles were added for 2 h according to the manufacturer’s instructions (CytoSelect 96-well phagocytosis assay, CellBiolabs, USA). Absorbance at 540 nm was measured using the Varioskan Lux Multimode microplate reader (Thermo Fisher Scientific).

### Nitric Oxide (NO) Production

To examine the level of NO production, Griess assay was performed. RAW264.7 macrophage cells were seeded into 96-well plates at a density of 4 × 10^4^ cells/well for 24 h. After incubation, LBB were added. After 22 h, supernatants were moved to new 96-well plates and 100 μl of Griess reagent (0.1% N-(1-naphthyl)ethylenediamine dihydrochloride, 1% sulfanilamide in 5% phosphoric acid) was added for the reaction. Plates were shaken for 1 min on an orbital shaker (Titramax 100, Heidolph Instruments GmbH & Co. KG, Germany) and incubated at RT for 15 min. Absorbance at 540 nm was measured using the Varioskan Lux Multimode microplate reader (Thermo Fisher Scientific). Nitrite concentrations were estimated using a nitrite standard curve.

### Immunoblot Analysis

RAW264.7 macrophage cells were seeded at a density of 2.25 × 10^6^ cells/well for 24 h. Then, LBB was treated to each well for 40 min and cells were washed with cold PBS. Cells were scraped off and collected in RIPA lysis buffer containing protease and phosphatase inhibitor cocktail (Sigma–Aldrich, USA). After centrifugation of the lysate, the protein concentration in supernatants was measured using the Pierce BCA Protein Assay Kit (Thermo Fisher Scientific). Proteins lysates were subjected to 10% SDS-PAGE and electrophoretically transferred to a nitrocellulose membrane (Bio-Rad, USA). After blotting, the membrane was blocked in 5% skim milk in TBS containing 0.1% Tween 20 (TBST) for 1 h, followed by the incubation in a specific primary antibody overnight at 4°C. Then, the membrane was rinsed with TBST and incubated with HRP-conjugated secondary antibody for 1 h. The membrane was visualized using Western Lightning Plus-ECL reagents (PerkinElmer, USA).

### Splenocytes Proliferation Assay

Mouse spleen was isolated from 6-week-old female C57BL/6 mice purchased from Young Bio (Republic of Korea). The animal studies were approved by the Institutional Animal Care and Use Committee (SNU-170220-2- 2) of Seoul National University, Seoul, Korea. Splenocytes were surgically isolated and minced by a 40 μm cell strainer in RPMI1640 containing 10% fetal bovine serum (Gibco) and 1% penicillin and streptomycin (Corning). The red blood cells in splenocytes were lysed by ACK lysis buffer (Gibco) on ice for 3 min. Then, cells were centrifugated and seeded into 96-well white luminescence plates. After that, LBB (10 μg/ml), and paclitaxel (10 μM) were treated to each well for 24 h. Then, the luminescences were measured as described in the *Cell viability assay* section.

### Bone Marrow-Derived Macrophages (BMDMs) Isolation

BMDMs were isolated from 6-week-old female C57BL/6 mice purchased from Young Bio . The animal studies were approved by the Institutional Animal Care and Use Committee (SNU-170220-2-2) of Seoul National University, Seoul, Korea. Bone marrow cells (BMCs) were isolated by flushing the femurs and tibias. Then, isolated BMCs were incubated with ACK lysis buffer (Gibco) on ice for 3 min to remove red blood cells. Then, BMDMs were seeded into 24 well plates in in DMEM/F-12 (Corning), containing FBS, 1% penicillin and streptomycin, and stimulated by 40 ng/ml M-CSF (PeproTech, USA) for 6 days.

### Animal Experiment

Male 5-week-old ICR were purchased from of Southeast Medi-Chem Institute (Republic of Korea). All experimental protocols were approved by the Institutional Animal Care and Use Committee (SEMI-19-01) of Southeast Medi-Chem Institute, Busan, Korea. Mice were accommodated for 1 week before the experiment and housed with a 12 h light/dark cycle in an air-conditioned room (23 ± 2°C). Mice were under *ad libitum* feeding condition including food and water. Mice were divided into two groups: (i) vehicle (PBS) only; (ii) LBB (1 × 10^12^ CFU/kg body weight). From day 0, every other day for 14 days, vehicle (PBS) or LBB was administered to mice orally through a metal gastric zonde. Food, water intake, and body weight were monitored at 3 day intervals and there was no significant difference between the control group and the LBB-treated group. On day 15, mice were anesthetized with CO_2_ and sacrificed for analysis of TNF-α production and splenocyte proliferation. Experiment design is described in Fig. 6.

### Statistical Analysis

Statistical analyses were performed using Student’s *t*-test, and *p*-values of less than 0.05 were considered statistically significant. The data were statistically analyzed using SPSS software (SPSS Inc., USA).

## Results

### LBB Treatment Promotes Activation of RAW264.7 Macrophage Cells

**Fig. 1:**
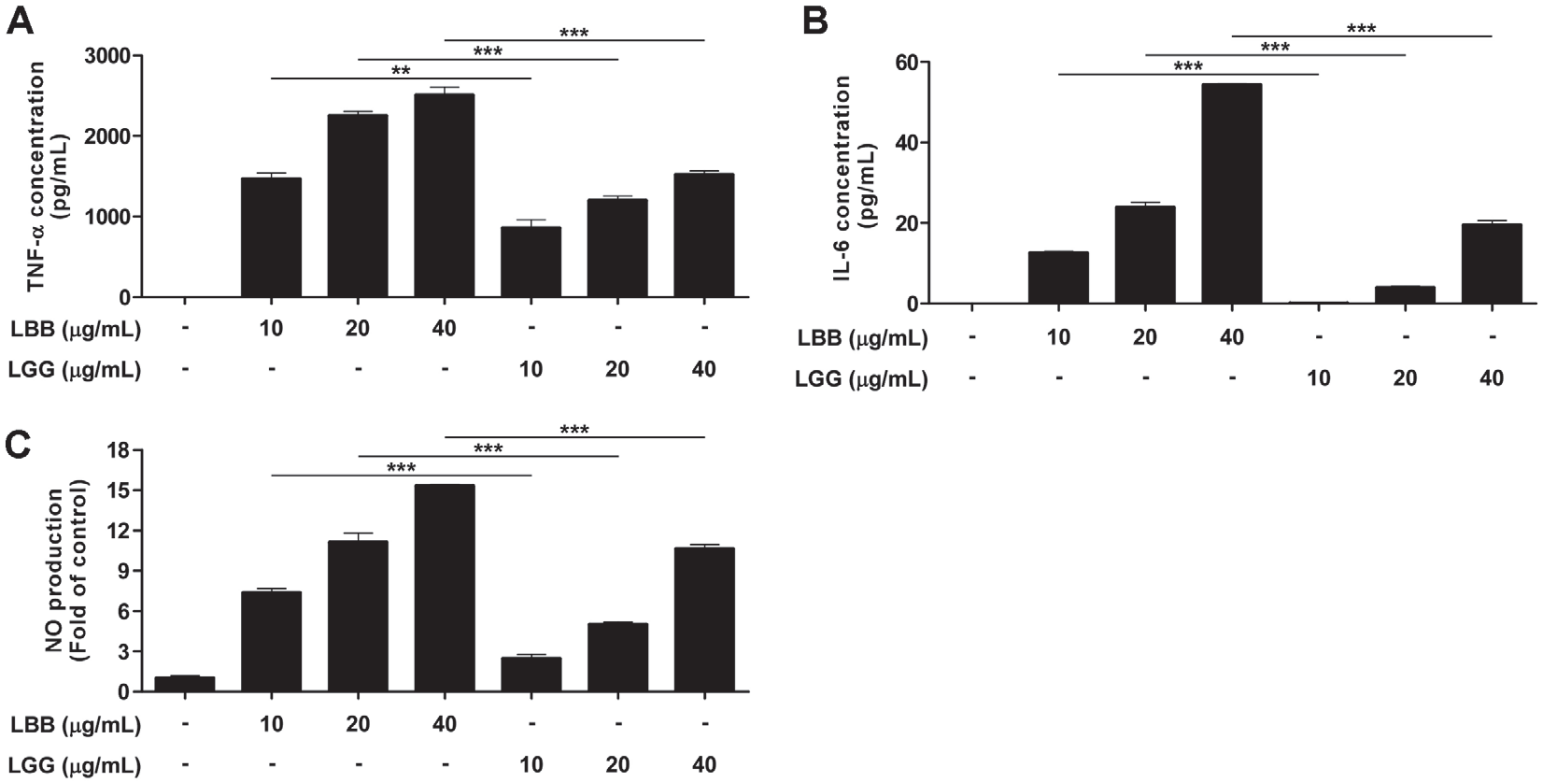
The effect of LBB on TNF-α, IL-6, and NO production. (**A**, **B**) RAW264.7 macrophage cells were treated with LBB or LGG at 10, 20, and 40 μg/ml, and the media was collected after 6 h. Cytokines were measured by ELISA. (**C**) RAW264.7 macrophage cells were treated with LBB and LGG at 10, 20, and 40 μg/ml. Media was harvested for Griess assay 22 h after LBB and LGG treatment. **p < 0.01, ***p < 0.001, significant difference between LBB treated group and LGG treated group (*n* = 3).

**Fig. 2:**
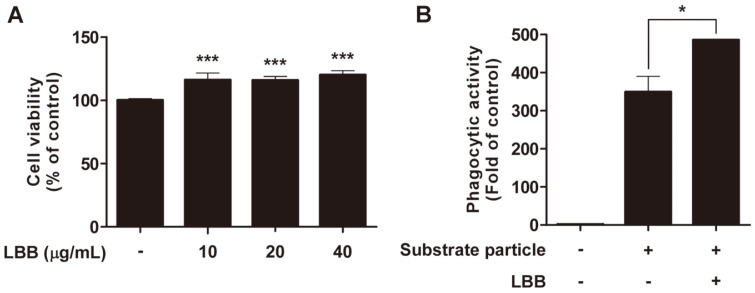
The effect of LBB on cell viability and phagocytosis activity in RAW264.7 macrophage cells. (**A**) RAW264.7 macrophage cells were treated with LBB at 10, 20, and 40 μg/ml for 24 h. ***p < 0.001, significant difference between LBB treated group and control (*n* = 3). (**B**) RAW264.7 macrophage cells were treated with LBB at 20 μg/ml and phagocytic activity was measured. Substrate particles; *E. coli*. *p < 0.05, significant difference between LBB treated group and the vehicle- only treated control group (*n* = 3).

We first tested whether LBB could affect the activity of macrophages using RAW264.7 cells. TNF-α and IL-6 were used as biomarkers to examine the immune-stimulatory effect of LBB [19]. Treatment with LBB led to a significant increase in TNF-α and IL-6 expression in RAW264.7 macrophage cells (Figs. 1A and 1B). Production of nitric oxide (NO) is important for macrophages to perform its anti-microbial and anti-tumorigenic activity [33]. LBB dose-dependently induced NO levels (Fig. 1C). Of note, the immune-stimulatory effect of LBB was stronger than that of another *L. brevis* strain (*i.e*.*, Lactobacillus brevis* KACC 11433) and LGG, a commonly used probiotic strain [34], further demonstrating the potency of LBB (Figs. 1A-1C and S1). In addition, LBB treatment did not show any cytotoxicity at the concentrations tested (Fig. 2A).

### LBB Enhances Phagocytic Activity of Macrophages

As phagocytosis is one of the major features of macrophages to defend against foreign pathogens [18], we investigated whether LBB treatment had an impact on the phagocytic activity. Incubation with *E. coli* particles induced phagocytosis and adding LBB significantly increased the phagocytic activity in macrophages (Fig. 2B).

### LBB Targets the TAK1 Signaling Pathway

**Fig. 3:**
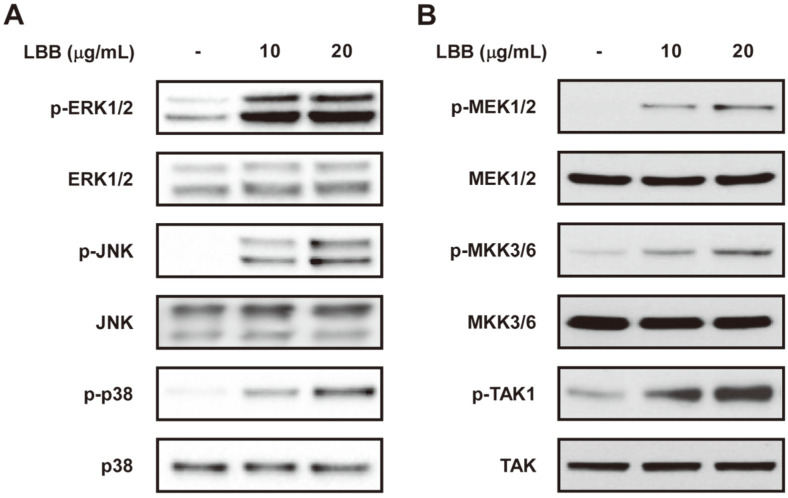
Involvement of TAK1, MAP2K, MAPK in the immunomodulatory effect of LBB. RAW264.7 macrophage cells were treated with LBB (10 or 20 μg/ml) for 40 min. Cell lysates were collected and subjected for immunoblotting using the corresponding antibody. Vinculin was used as a loading control.

To elucidate the molecular mechanism responsible for the immunomodulatory effect observed after LBB treatment, we first analyzed the MAPK pathway. ERK, p38, and JNK signal transduction pathways have been reported to be involved in the regulation of macrophage activation [22]. LBB treatment significantly induced phosphorylations of ERK, p38, and JNK (Fig. 3A). Next, we further examined the upstream regulators of MAPK. LBB was able to cause an upregulation of MEK1/2 and MKK3/6 activity (Fig. 3B). TAK1 is a crucial player in the control of immune responses and conveys its signals through the MAP2K-MAPK pathway [35, 36]. Interestingly, phosphorylation of TAK1 was induced by LBB treatment. These results suggest that LBB activates the TAK1- MAP2K-MAPK signaling axis to promote immune stimulation.

### LBB Exerts Immune-Stimulatory Effects in Primary Immune Cells

**Fig. 4:**
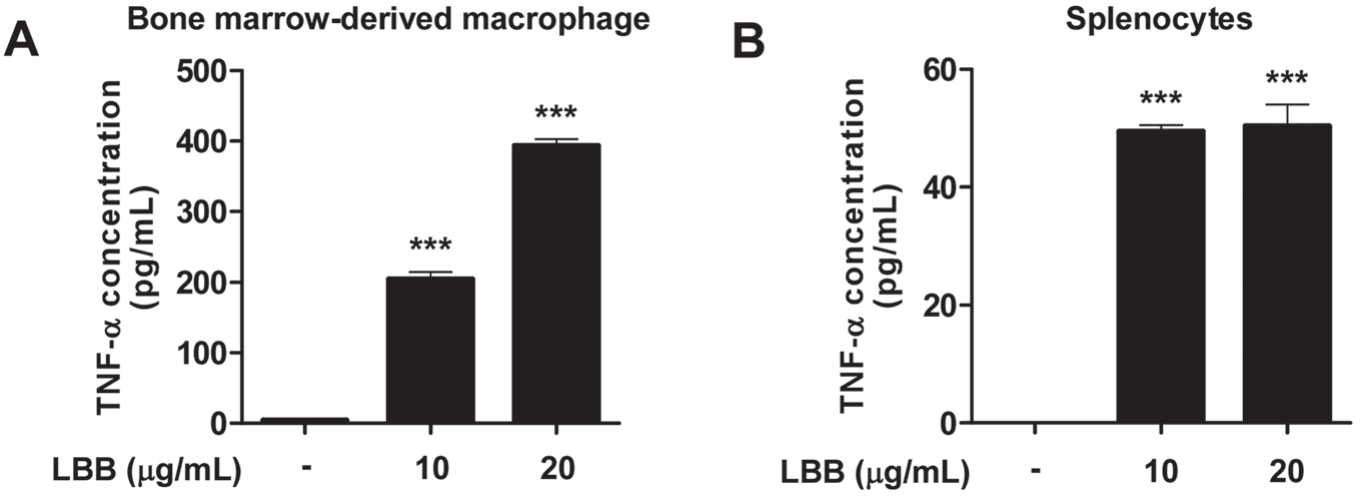
LBB induces TNF-α in primary BMDMs and splenocytes. (**A**) BMDMs were isolated from C57BL/6 mice and differentiated for 6 days. Media was harvested 6 h after LBB treatment for TNF-α analysis. (**B**) Splenocytes were isolated from C57BL/6 mice. Media was harvested after LBB treatment for 18 h for TNF-α analysis. ***p < 0.001, significant difference between LBB treated group and control (*n* = 3).

We next investigated if the immune-stimulatory activity of LBB could be recapitulated in primary immune cells. Bone marrow-derived macrophages (BMDMs) were isolated from mice and subjected to LBB treatment. LBB was able to increase TNF-α production in BMDMs (Fig. 4A). LBB also induced TNF-α in primary splenocytes (Fig. 4B), demonstrating that the effect of LBB can be observed in primary immune cells.

**Fig. 5:**
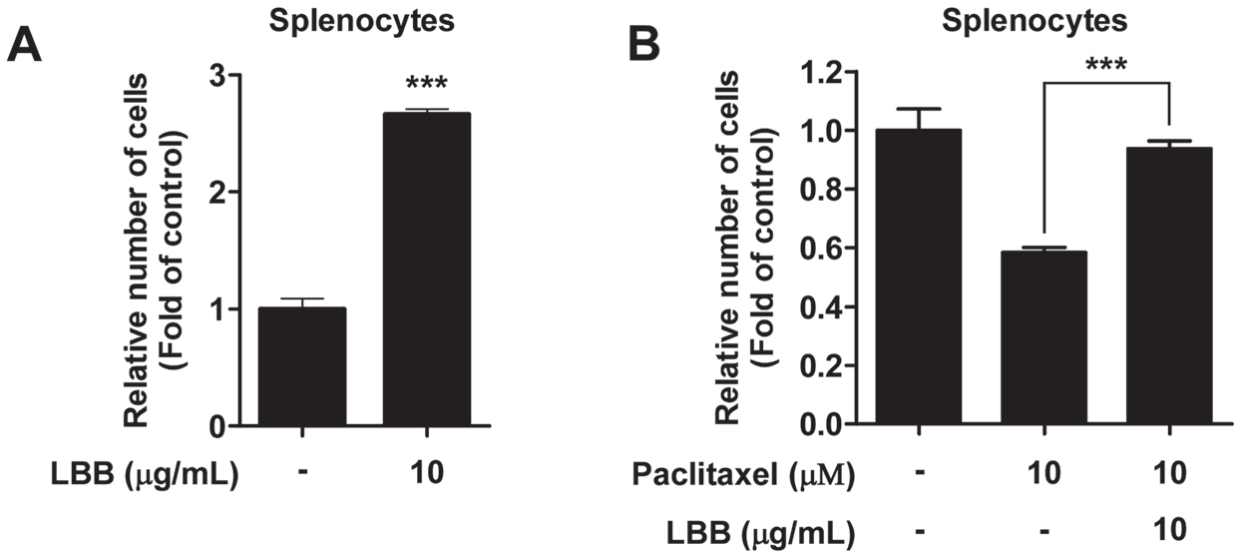
The effect of LBB on primary splenocytes proliferation. Splenocytes were isolated from C57BL/6 mice. Cell viability assays were conducted (**A**) 72 h and (**B**) 24 h after LBB treatment. ***p < 0.001, significant difference between LBB treated group and control (*n* = 3).

Proliferation of immune cells are a hallmark of immune activation [37]. Interestingly, we also found that LBB treatment enhanced the proliferation of splenocytes (Fig. 5A). Some cancer chemotherapeutic drugs have been reported to show cytotoxic effects towards immune cells, generating undesirable immunosuppressive side effects to patients [38]. As LBB promoted the proliferation of splenocytes, we examined if the effect of LBB could be extended to improve the cell viability of immune cells under drug-mediated cell death conditions. LBB treatment displayed protective effects against paclitaxel-induced reduction in cell viability (Fig. 5B).

### LBB Generates Immune-Stimulatory Activity In Vivo

**Fig. 6:**
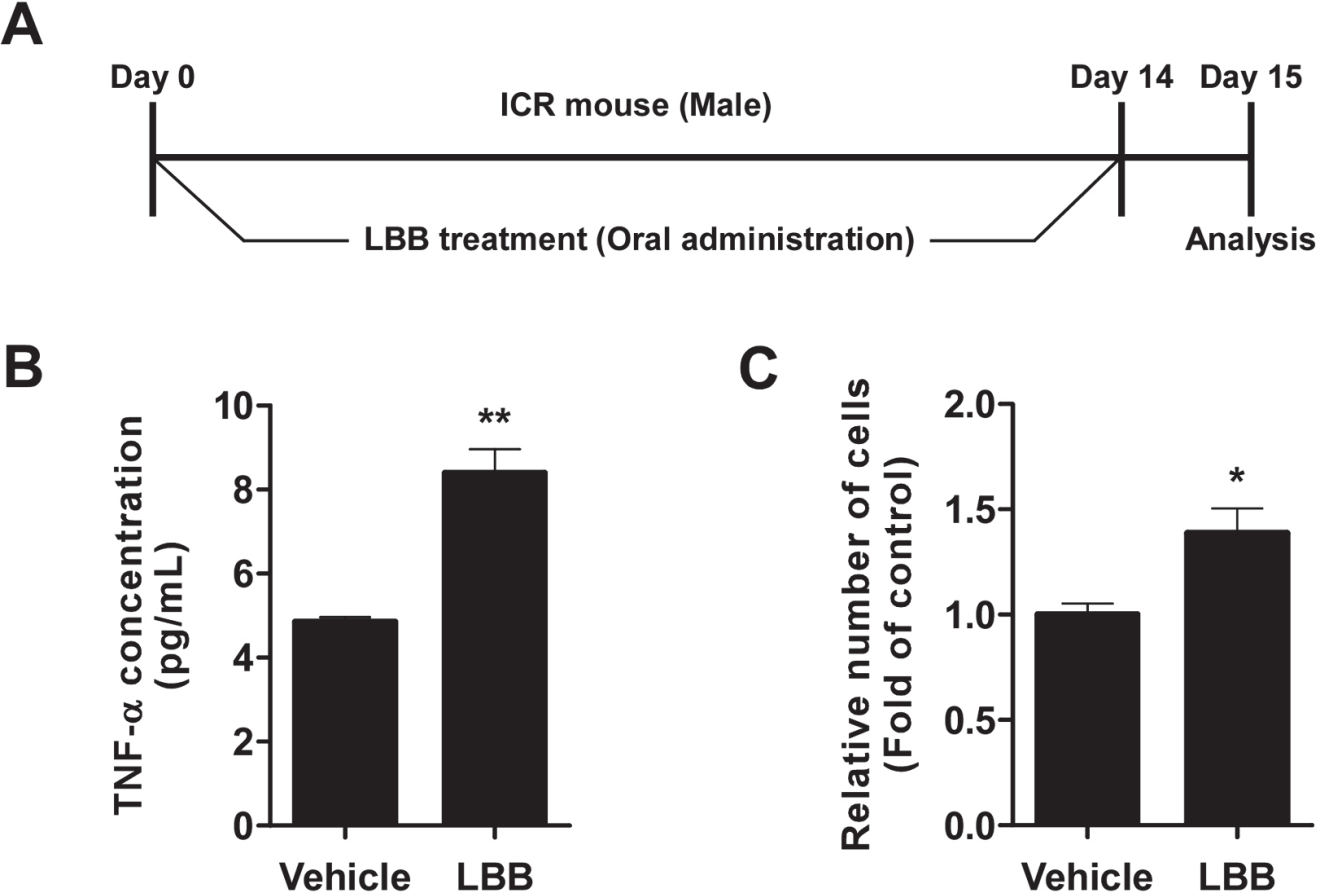
The immune-stimulatory effect after oral administration of LBB to ICR mice. (A) Mice were orally administrated with LBB for 14 days. (**A**) Mice were orally administrated with LBB for 14 days. (**B**, **C**) Splenocytes were isolated from ICR mice. Splenocytes were seeded and used for further analysis. Media was harvested 72 h after the isolated cells were seeded for TNF-α analysis. Celltiter-glo luminescent cell viability assay was conducted 72 h after the isolated cells were seeded. The concentration of LBB treatments was 1 × 10^12^ CFU/kg body weight. *p < 0.05, **p < 0.01, significant difference between LBB treated group and the vehicle-only treated control group (*n* = 3).

To further confirm the efficacy of LBB, we administered LBB to mice and examined whether the immune- stimulatory effects can be translated in vivo. LBB was administered orally for 14 days and the splenocytes were isolated from mice (Fig. 6A). Splenocytes from mice consuming LBB showed higher expression of TNF-α compared to the control group (Fig. 6B). Mice administered with LBB exhibited greater proliferative rate compared to vehicle treated mice (Fig. 6C).

## Discussion

Probiotics have been recognized to produce various health-beneficial properties in humans. Although live cultures are more commonly used for oral administration, recent studies have suggested that non-viable probiotics can also generate bioactivities and may have some advantages over viable probiotics [39, 40]. Consuming heat-killed probiotics are believed to be safer than live probiotic cultures [41]. Viable probiotics can move from the gut to other organs [42], cause infections [43], and induce antibiotic resistances [44]. On the other hand, heat-killed probiotics have lower risks for antibiotic resistances compared to viable probiotics [40]. Probiotics that are inactivated by heat treatment can also provide commercial benefits in terms of prolonged shelf life, as well as reducing restrictions for shipping and storage due to lower reactions with other ingredients [45]. Thus, identifying probiotic strains with potential health benefits after inactivation can be useful for the development of novel functional foods or nutraceuticals.

In the current study, we inactivated *L. brevis* KCTC 12777BP by heat-treatment at 121°C and investigated its immune-stimulatory effects in RAW264.7 macrophage cells, primary macrophages and splenocytes, and mice. We found that LBB increases TNF-α and IL-6 production in macrophages and splenocytes. LBB also elevated NO secretion which endows macrophages to block pathogen replication[46]. Notably, LBB exhibited higher stimulatory activity towards the secretion of cytokines and NO compared to that of LGG, a well-known immunomodulatory probiotics strain [47, 48]. Multiple studies have shown that even within the same species, different strains can show significanly different bioactivities [49-51]. In order to prove this concept we have performed an experiment using another *L. brevis* strain (*i.e*., *L. brevis* KACC 11433) in comparison with *L. brevis* KCTC 12777BP (Fig. S1). Both bacteria were prepared using identical methods and treated to RAW264.7 macrophage cells. Indeed, we have found that *L. brevis* KCTC 12777BP (LBB) and *L. brevis* KACC 11433 displayed significantly different results regarding TNF-alpha production. This could at least partially explain the result from a previous study where *L. brevis* KCCM 12203P showed relatively lower TNF-α secretion levels compared to that of LGG [52]. We also observed that LBB can enhance phagocytosis of *E. coli* particles in macrophages. This is the first study to report the phagocytosis-stimulatory effect of heat-killed *L. brevis*. In the in vivo study, splenocytes isolated from LBB-administered mice displayed higher proliferative capacaity and TNF-α secretion levels compared to splenocytes from vehicle-administered mice. In the current study, we have used heat-killed whole cell lysates of LBB. The purpose of the study was to identify the bioactivity LBB which could be potentially used as a food material. Thus, considering the practical application of LBB in the food industry we have chosen to use the whole cell lysates. According to a previous study [53], both the cell wall and the cytoplasmic portion from various types of probiotics displayed bioactivity in RAW264.7 macrophage cells. Although a more detailed further analysis would be required, based on this previous study, we also speculate that the cell wall as well as the cytoplasmic portion of LBB would exert bioactivity.

In addition to macrophages, we utilized splenocytes to examine the immune-stimulatory effect of LBB. We treated LBB in vitro against isolated primary splenocytes and observed immune-stimulatory results. We also assessed splenocyte proliferation and TNF-α secretion after orally administering LBB to mice. Spleen is a major organ harboing diverse types of immune cells and the inhabiting cells are crucial for executing proper immune responses in the body [54]. Splenocytes contain diverse types of immune cells including macrophages, dendritic cells, natural killer cells, T cells, and B cells [55]. Increase in the proliferation rate and cytokine levels from splenocytes after LBB treatment implies stimulation of immunity which may not only be restricted to macrophages but also to other type of immune cells [56, 57]. The potential systemic immune response seen after administering LBB in vitro and in vivo suggests the possibility for further developing LBB as an immune enhancing agent.

**Fig. 7:**
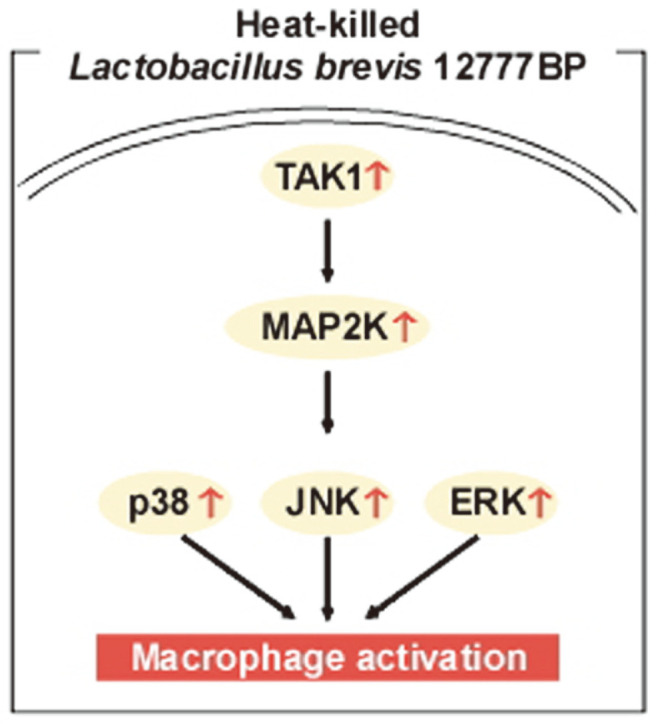
Immune-stimulatory activity of LBB. The possible mode of action is summarized.

LBB dose-dependently upregulated the MAPK signaling pathway including ERK, JNK, and p38. Previous studies have demonstrated that ERK controls the transcription and translocation of TNF-α [58, 59], and JNK and p38 contribute in regulating both mRNA stability and transcription of TNF-α [60, 61]. Interestingly, LBB also increased the phosphorylation of MAP2K (*i.e*., MEK1/2 and MKK3/6), the upstream regulators of MAPKs. And more importantly LBB treatment led to a strong activation of TAK1 which is a member of the MAP3K family [20] (Fig. 7). TAK1 has been implicated to play a major role in controlling immune responses and its activation contributes to proper functioning of the immune defense system [62]. It is likely that the immune-stimulatory effect of LBB involves the activation of TAK1 and its downstream MAPK family members.

This study is the first to report that heat-killed *Lactobacillus brevis* can enhance the phagocytic activity against *E. coli* particles and stimulate immune responses. In addition, we found LBB can more potently induce immunomodulatory effects compared to that of LGG. There is a great interest for identifying novel probiotics with immunomodulatory function. These results suggest that LBB can be used as a potent immune-stimulatory agent and be further studied for development as a nutraceutical.

## Supplemental Materials



Supplementary data for this paper are available on-line only at http://jmb.or.kr.
