# Supplementary material for: Heat-Killed Lactobacillus brevis Enhances Phagocytic Activity and Generates Immune-Stimulatory Effects through Activating the TAK1 Pathway
Source: J Microbiol Biotechnol. 2020 Jul 19;30(9):1395–403. doi: 10.4014/jmb.2002.02004 (PMC9728231; doi:10.4014/jmb.2002.02004)
Supplement: Supplementary file 1 [file JMB-30-9-1395-supple.pdf]

J. Microbiol. Biotechnol.
https://doi.org/10.4014/jmb.2002.02004

Table S1. Identification of *Lactobacillus brevis* KCTC 12777BP

16S rRNA identification	Similarity (%)
Lactobacillus brevis	99.86

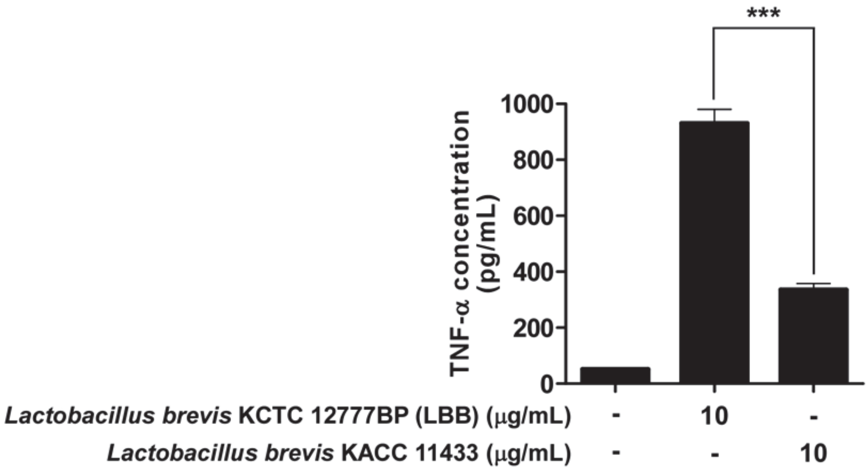

Figure S1. The effect of LBB and *Lactobacillus brevis* KACC 11433 on TNF-α production in RAW264.7 macrophage cells. RAW264.7 macrophage cells were treated with LBB or *Lactobacillus brevis* KACC 11433 at 10 μg/mL, and the media was collected after 6 hr. Cytokines were measured by ELISA. ***p<0.001, significant difference between LBB treated group and *Lactobacillus brevis* KACC 11433 treated group (n=3).
